# Modeling and optimizing in vitro percentage and speed callus induction of carrot via Multilayer Perceptron-Single point discrete GA and radial basis function

**DOI:** 10.1186/s12896-022-00764-4

**Published:** 2022-11-05

**Authors:** Masoumeh Fallah Ziarani, Masoud Tohidfar, Mohammad Navvabi

**Affiliations:** 1 Department of Plant Biotechnology and Life Science, Shahid Beheshti University, Tehran, Iran; 2 Department: Space Engineering, Faculty: New Technologies and Energy Engineering, Shahid Beheshti University, Tehran, Iran

**Keywords:** Artificial neural network, Perceptron-Single point discrete GA model, Micropropagation, Carrot

## Abstract

**Background:**

Callus induction is the first step in optimizing plant regeneration. Fit embryogenesis and shooting rely on callus induction. In addition, using artificial intelligence models in combination with an algorithm can be helpful in the optimization of in vitro culture. The present study aimed to evaluate the percentage and speed of callus induction optimization in carrot with a Multilayer Perceptron-Single point discrete genetic algorithm (GA).

**Materials and methods:**

In this study, the outputs included callus induction percentage and speed, while inputs were different types and concentrations of plant growth regulator (0. 5, 0.2 mg/l 2,4-D, 0.3, 0.2, 0.5 mg/l BAP, 1, 0.2 mg/l Kin, and 2 mg/l NAA), different explants (shoot, root, leaf, and nodal), a different concentration compound of MS medium (1 × MS, 4× MS, and 8× MS) and time of sampling. The data were obtained in the laboratory, and multilayer perceptron (MLP) and radial basis function (RBF), two well-known ANNs, were employed to model. Then, GA was used for optimization, and sensitivity analysis was performed to indicate the inputs’ importance.

**Results:**

The results showed that MLP had better prediction efficiency than RBF. Based on the results, R^2^ in training and testing data was 95 and 95% for the percentage of callus induction, while it was 94 and 95% for the speed of callus induction, respectively. In addition, a concentration compound of MS had high sensitivity, while times of sampling had low sensitivity. Based on the MLP-Single point discrete GA, the best results were obtained for shoot explants, 1× MS media, and 0.5 mg/l 2, 4-D + 0.5 mg/l BAP. Further, a non-significant difference was observed between the test result and predicted MLP.

**Conclusions:**

Generally, MLP-Single point discrete GA is considered a potent tool for predicting treatment and fit model results used in plant tissue culture and selecting the best medium for callus induction.

**Supplementary Information:**

The online version contains supplementary material available at 10.1186/s12896-022-00764-4.

## Introduction

Today, genetic engineering is used for different goals in plants, including improving, modifying, or creating a new trait and producing vaccines by molecular farming [1]. For this purpose, optimizing plant tissue culture is crucial. The first step in tissue culture produces high and fit callus induction.

The carrot is a plant model for producing vaccines by molecular farming [1]. For this reason, optimizing the tissue culture of carrots is essential. The best callus is the first step for proper regeneration and healthy plantlets [2–4].

Many studies were conducted to improve callus induction [5, 6]. In this regard, different explants, plant growth regulators, and media cultures were used for callogenesis. Testing all of these treatments is costly and time-consuming.

Seemingly, new methods are required for modeling and optimizing callus induction, while traditional models may not be suitable. Several non-linear biological processes based on Artificial Neural Network (ANN) can be used for this purpose since they have more flexibility in modeling in vitro culture [7–9]. Different types of ANNs are available such as Generalized Regression Neural Network (GRNN), Multilayer Perceptron (MLP), radial basis function (RBF), and Probabilistic Neural Network (PNN) [10]. Many studies used the ANN model for modeling and optimizing plant tissue culture [7, 11, 12]. However, the ANN model has not formerly been used to optimize callus induction in carrot plant. Thus, the present study is considered the first report of in vitro callus induction model in the carrot.

MLP is a type of ANNs, applied for different purposes, such as clustering, predicting, and classifying complex systems [13]. MLP can identify the relationship between target and input variables and recognize the inherent knowledge in the datasets without previous physical considerations [14]. However, MLP does not present a neat mathematical formula that illustrates the relative relationship of each independent variable in the model. Hence, MLP is considered a “black box.” MLP consists of numerous highly interconnected processing neurons working parallel to solve a particular problem [14]. MLP is learned by example. The examples should be carefully chosen; otherwise, time is wasted, or even worse, the model might work inaccurately [14]. The main demerit point of MLP is that its operation can be unpredictable because MLP learns how to find the solutions by itself [14]. On the other hand, RBF is engaging, powerful, and easy-to-interpret ANNs with supervised learning [15]. However, most plant tissue culture studies have employed the individual ANN, and the comparison between these ANNs has rarely been studied in the tissue culture area [16].

Multi-objective functions can improve optimization problems in plant tissue culture and callus induction. In addition,many trials and errors to optimize the input data. Today, researchers have implemented a Genetic Algorithm (GA) to reduce the volumes of the calculations [7, 10, 11]. Many parameters should be considered for optimizing the callus induction. The GA is unsuitable for this purpose since it cannot check several factors simultaneously [17, 18]. Non-dominated Single point discrete GA was used for the first time to solve this problem, considered the first algorithm for evolutionary multi-objective optimization. In addition, it can analyze multiple parameters simultaneously [19].

Different machine learning algorithms (e.g., ANNs, neuro-fuzzy logic systems, support vector machine (SVM), and random forest) have been recently used for modeling and predicting various in vitro culture systems such as explant sterilization [13, 20], in vitro seed germination [21], callogenesis [22–24], androgenesis [25], shoot proliferation [15, 26], rhizogenesis [27], in vitro secondary metabolite production [28–30], and gene transformation [31, 32]. Among machine learning algorithms, different types of ANNs, such as MLP, radial basis function (RBF), and generalized regression neural network (GRNN), have been widely employed to model and predict in vitro culture processes [16, 33, 34]. Patra et al. used an ANN-genetic algorithm and response surface methodology to optimize ultrasound-assisted extraction of ascorbic acid, protein, and total antioxidants from cashew apple bagasse using an ANN-genetic algorithm and response surface methodology [35]. The application of ANNs and GAs to predict and optimize greenhouse banana fruit yield through nitrogen, potassium, and magnesium was performed by Ramezanpour et al. [36]. Also, artificial neural networks and genetic algorithm used for optimization in food processing [37].

For the first time, the present study aims to find the best optimization for a type of callus induction of some parameters such as explants (nodal, root, leaf, and shoot), sampling day, and type and concentration of plant growth regulator. Besides, the concentration of Murashige and Skoog (MS) medium was performed using non-linear MLP-non-dominated Single point discrete GA and radial basis function modeling in carrot.

## Materials and methods

### Plant materials

Seeds of carrot (*Daucus carota* L. cv. Nantaise) were prepared from Seed and Plant Improvement Institute (Karaj, Iran). Then, the seed surface was sterilized with a solution of 70% ethanol for 1 min, sodium hypochlorite (5%) for 20 min, and rinsed with sterile water three times [38].

### Media and culture condition

The sterile seeds were cultured in ½ MS medium supplemented with 3% sucrose and solidified with 0.8% agar. Then, the pH of the medium was regulated to 5.7 by 1 N KOH or 1 N HCl before autoclaving, maintained at 25 ± 2 °C, and exposed to light for 16 h and dark for 8 h per day [38]. Ultimately, the seedling was prepared after two to three weeks of culturing sterile seeds [39].

### Experimental design

Foremost, the explants were used, including root, shoot, nodal, and leaf. Then, they were excised in 3-mm pieces. The experiments were performed based on a Completely Randomized Design (CRD) with 24 replicates per treatment by three sub-sets. The data were analyzed using SPSS software, and different means were identified using Tukey’s test (*P* = 0.05).

The eight treatments were evaluated for callus induction in carrot. In this study, the type and concentration of plant growth regulators [2, 4-Dichlorophenoxyacetic acid (2, 4-D), 6-Benzylaminopurine (BAP), Kinetin (Kin), and 1-Naphthaleneacetic acid (NAA)], different concentrations of MS medium (1× MS, 4× MS, 8× MS) used for different explants (leaf, nodal, root, and shoot), different times of sampling (8 and 25 days) were analyzed. Table 1 shows the results of data analysis.

**Table 1:** Statistics of MLP models for the percentage of callus induction and speed of callus induction of carrot (training vs. testing values) in carrot

Item	Percentage of callus induction		Speed of callus induction	
	Training	Testing	Training	Testing
R Squar	0.95	0.95	0.94	0.95
RMSE	134.66	451	8.12	389

The reason for choosing 4× MS and 8× MS in this research was to investigate the effect of increasing minerals on the speed and percentage of callus induction.

In this research, hormonal compounds were used from previous studies, as well as new hormonal compounds with different concentrations, to determine the best hormonal combination to reach the highest speed and percentage of callus formation.

The effect of different treatments on callus induction was evaluated after 25 days of culture. The obtained data from laboratory tests were used for modeling and optimization using MLP-non-dominated Single point discrete GA (data not shown).

### Calculatingthe speed of callus induction

Speed of callus induction obtained from sampling time divided by average callus diameter.

### Multilayer Perceptron (MLP) model

**Fig. 1:**
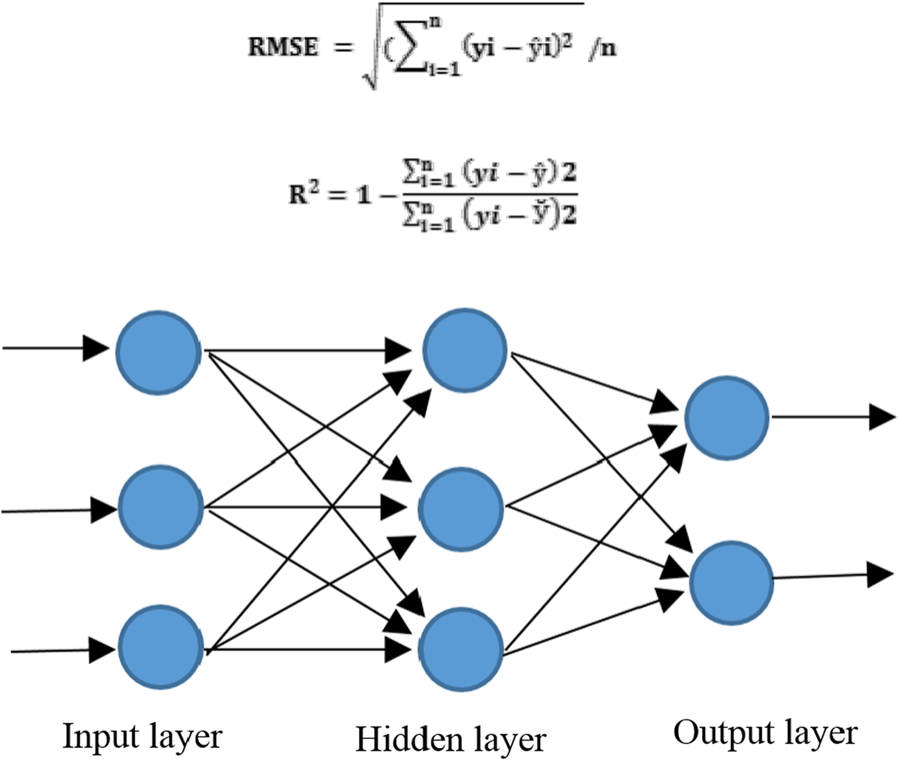
The schematic plan of the proposed MLP method

The type and concentration of plant growth regulators (2, 4-D, BAP, Kin, and NAA), different concentrations of MS medium (1× MS, 4× MS, 8× MS) used for different explants (leaf, nodal, root, and shoot), and different times of sampling (8 and 25 days) were considered the inputs. In addition, the speed and percentage of callus induction were considered the outputs in the MLP model (Fig. 1). In this study, 90% of the data were used for training and 10% for testing the data in the model. Different values of the parameters for a significant model were used to indicate the best construct of each model based on error analysis. Lastly, in each model, the results were obtained by considering the minimum estimation error based on Root Mean Square Error (RMSE) and the coefficient of determination (R^2^) as follows:

$${\text{RMSE}} = \sqrt {\mathop \sum \limits_{{{\text{i}} = 1}}^{{\text{n}}} (yi - \hat{y}i)^{2 } } /n$$

$${\text{R}}^{2} = 1 - \frac{{\mathop \sum \nolimits_{{{\text{i}} = 1 }}^{{\text{n}}} \left( {yi - \hat{y}} \right)2}}{{\mathop \sum \nolimits_{{{\text{i}} = 1 }}^{{\text{n}}} \left( {yi - \overset{\lower0.5em\hbox{$\smash{\scriptscriptstyle\smile}$}}{y} } \right)2}}$$

As shown above, the best fit for RMSE and R^2^ values is 0 and 1, respectively. The MLP includes an input layer, one or more hidden layers, and an output layer [40, 41]. In MLP, the number of hidden layers is shown along with the number of neurons in each layer [40].

In this study, Multilayer Perceptron-Single point discrete GA was used with 11 hidden layers by Error Back Propagation (EBP) algorithm with a Bayesian method to indicate the speed and percentage of callus induction. The proper response of this method in a nonlinear model and sustainability in the face of noisy data are considered the main reasons for using Multilayer Perceptron-Artificial Neural Network and Bayesian method [42–44].

### Optimization process with MLP

Chromosome/population value should be first given for evaluating decision variables, mutation, selection, and cross-over to select the best solution by MLP. Finally, the members with the highest rank should be removed, and the new members as parents should be selected for producing the next generation.

Obtaining the best fitness was used to select the best population. One hundred initial populations and 1000 repeats were set.

The optimal input values included different concentrations of 2, 4-D, BAP, Kin, and NAA, different concentrations of MS, and various explants such as the leaf, nodal, root, and shoot. Different sampling times were determined to achieve the best output speed for callus induction based on the MLP Model.

### Radial basis function (RBF)

RBF is a three-layer ANN consisting of an input layer, a hidden layer, and an output layer. This is the basis and principle for radial basis networks, which organize statistical ANNs. Statistical ANNs refer to networks that, in contrast to the traditional ANNs, implement regression-based approaches and have not been emulated by biological neural networks [45]. In an RBF model, the Euclidean distance between the center of each neuron and the input is considered the input of the transfer function for that neuron. The most well-known transfer function in RBF is the Gaussian function, which is determined based on the following equation:

$$f(X_{r} ,X_{b} ) = e^{{ - [||X_{r} - X_{b} ||*0.8326/h]^{2} }}$$

where $X_{r}$, $X_{b}$, and *h* are input with unknown output, observed inputs in time *b*, and spread, respectively. The function’s output was close to 1 when $||X_{r} - X_{b} ||$ approached 0 and 0 when $||X_{r} - X_{b} ||$ approached a significant value. Finally, the dependent variable (*Yr*) by predictor *X*_*r*_ was determined as follows:

$$Y_{r} = \sum\limits_{b = 1}^{m} {w_{b} *f(X_{r} ,X_{b} ) + w_{0} }$$

where *w*_0_ and $w_{j}$ are the bias and weight of linkage between the *b*th hidden layer and the output layer, respectively.

### Sensitivity analyses

Sensitivity analysis was used to determine which input parameters affected the output model most. In addition, it was used to determine which input parameters, including the type and concentration of 2, 4-D, BAP, Kin, and NAA, different concentrations of MS medium (1× MS, 4× MS, 8× MS), different explants such as the leaf, nodal, root, and shoot, as well as different times of sampling (8 and 25 days) were effective most in obtaining the highest speed and percentage of callus induction. The sensitivity of output vs. input variables was determined based on the variable sensitivity error (VSE) value, which indicates the performance of the developed MLP model when all data are unavailable. Further, it specified a relative indication between the MLP model error and the VSE when all variables are available. VSR ranks the variables, where the essential variable has a higher value of VSR. The analysis indicated which variable is more significant than the other parameters for callus induction.

### Validation experiment

The obtained data and laboratory results, including the speed and percentage of callus induction, were used for optimization by MLP-Single point discrete GA and tested for evaluating the effects of the MLP-Single point discrete GA model. Then, the speed and percentage of callus induction were optimized as in vitro parameters.

### Statistical analysis

ANOVA analysis was performed based on a CRD with three replications. Tukey's test identified significantly different means (*P* = 0.05).

## Results

After analyzing the data obtained in the laboratory using SPSS software, the following results were obtained.

The purpose of this investigation was to compare the results obtained in the laboratory with the results obtained from the ANN.

### In MS medium (MS in natural concentration)

**Fig. 2:**
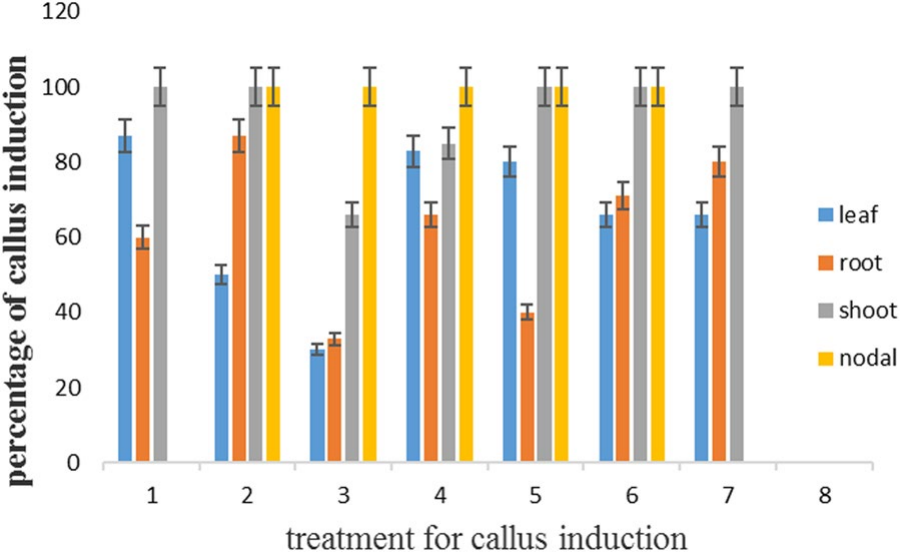
Percentage of callus induction in MS medium in carrot

Regarding MS medium in natural concentration in Additional file 1, the highest callus induction was obtained at shoot explants in media 1, 2, 5, 6, and 7, as well as nodal explants in media 2, 3, 4, 5, and 6. However, the lowest callus induction was reported in nodal explants in media 1, 7, and all explants in medium 8 (Fig. 2) (Fig. 6. A, B, C).

**Fig. 3:**
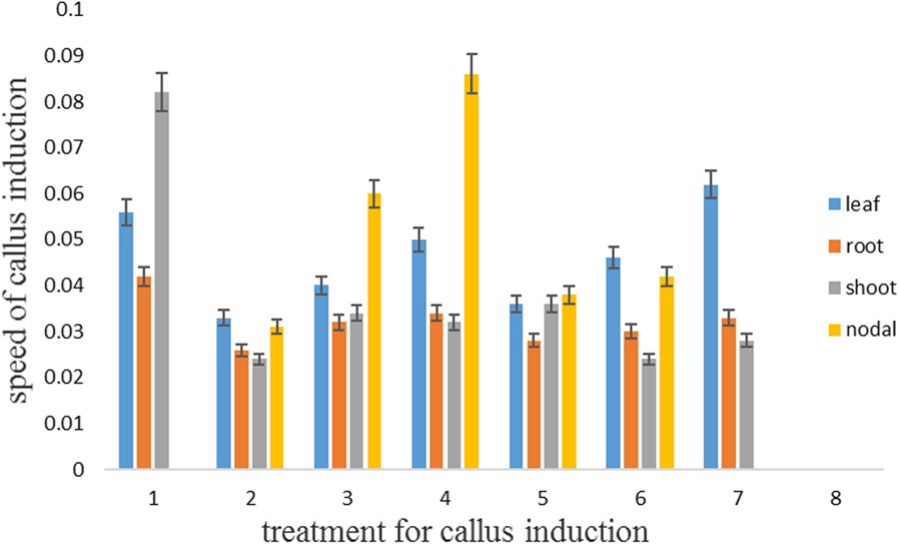
Speed of callus induction in MS medium in carrot

In addition, the maximum speed of the callus was observed in the leaf and root in medium 4. In contrast, the minimum speed of callus was reported in nodal explant in media 1 and 7 and all explants in medium 8 (Fig. 3).

### In 4× MS medium (MS in 4× concentration)

**Fig. 4:**
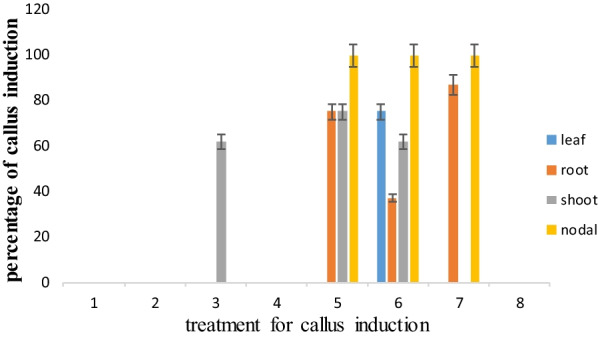
Percentage of callus induction in 4 MS medium in carrot

In 4× MS medium (MS in 4× concentration in Additional file 2), the percentage of callus induction in media 5, 6, and 7 was 100%, while it was 0% in media 1, 2, 4, and 8 (Fig. 4) (Fig. 6D, E).

**Fig. 5:**
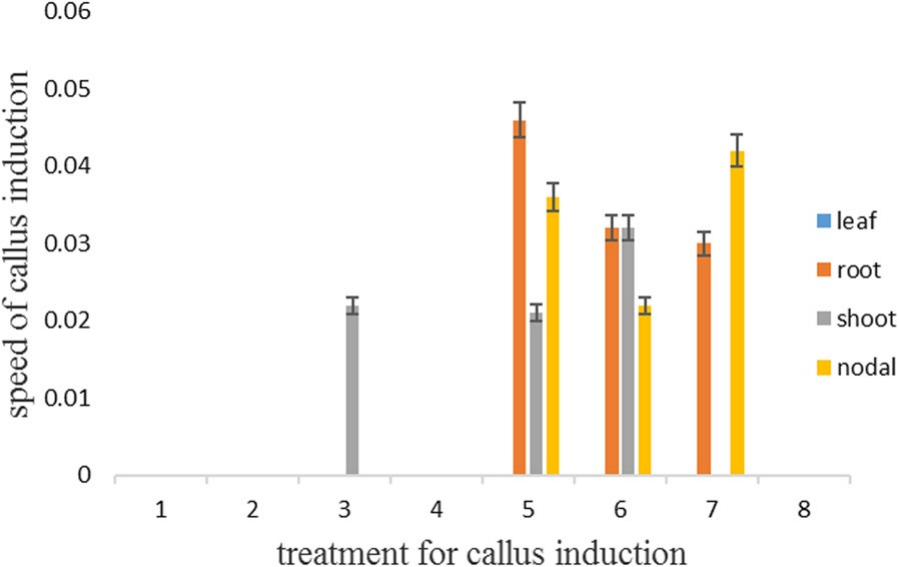
Speed of callus induction in 4 MS medium in carrot

Further, the highest speed of callus was in root explants in treatment 5, while the lowest was in 1, 2, 4, and 8 media in all explants (Fig. 5).

### In 8× MS medium (MS in 8× concentration)

**Fig. 6:**
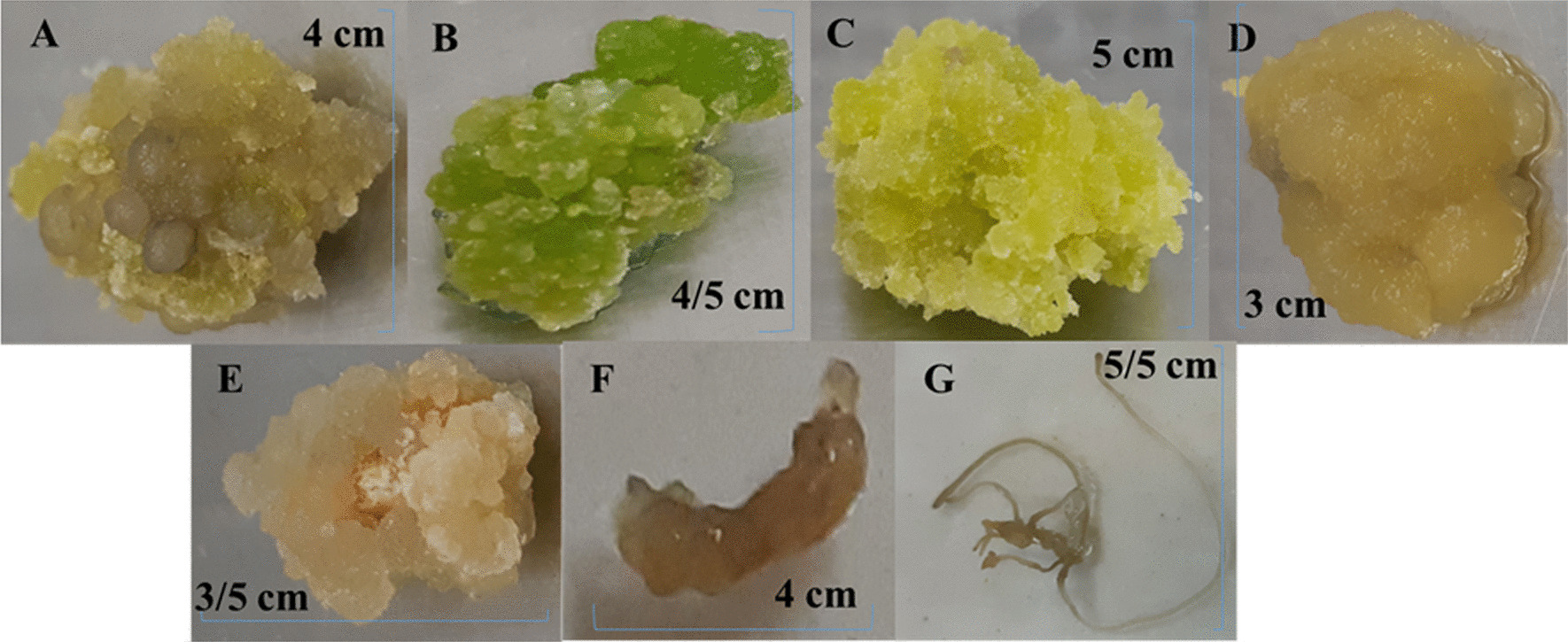
**A** Callus induction of root explant in MS medium of treatment 6. **B** Callus induction of leaf explant in MS medium of treatment 6. **C** Callus induction of shoot explant in MS medium of treatment 6. **D** Callus induction of leaf explant in 4 MS medium of treatment 6. **E** Callus induction of shoot explant in 4 MS medium of treatment 6. **F** Callus induction of shoot explant in 8 MS medium of treatment 6. **G** Callus induction of root explant in 8 MS medium of treatment 6

Regarding 8× MS medium (MS in 8× concentration in Additional file 3), the callus induction and speed of callus in all treatments and four explants were 0% (Fig. 6F, G).

The results indicated the best medium for percentage and speed callus induction was MS medium in treatment six by shoot explant. In 8× MS medium by all explants and treatments, the percentage and speed of callus induction was 0%.

Based on the obtained results, the type and concentration of explants and plant growth regulator, kind of explants and concentration of the compound in MS medium, and sampling time should be optimized for obtaining the highest callus induction. Data were also analyzed with the MLP model to confirm obtained results in the laboratory.

### Modeling and comparative analysis of MLP and RBF

MLP and RBF models were used to confirm the results obtained in the laboratory. In the current study, MLP and RBF models were used to model the speed and percentage of callus induction as an output on base input, including different types of explants and other kinds and concentrations of plant growth regulator, different concentrations of compounds in MS medium. Data modeling through machine learning algorithms can provide a reliable approach to improve detailed knowledge of carrot speed and the percentage of callus induction. Then, MLP Model was used to analyze the predicted and accurate data. R^2^ in training and testing data was over 95% and 95%, and 94% and 95% in the percentage and speed of callus induction by MLP model, respectively. By RBF model R^2^ in training and testing data were 88% and 83%, and 81% and 83% in the callus induction percentage and speed, respectively (Tables 1, 2).

**Table 2:** Statistics of RBF models for the percentage of callus induction and speed of callus induction of carrot (training vs. testing values) in carrot

Item	Percentage of callus induction		Speed of callus induction	
	Training	Testing	Training	Testing
R Squar	0.88	0.83	0.81	0.83
RMSE	140.55	480	10.12	401

**Fig. 7:**
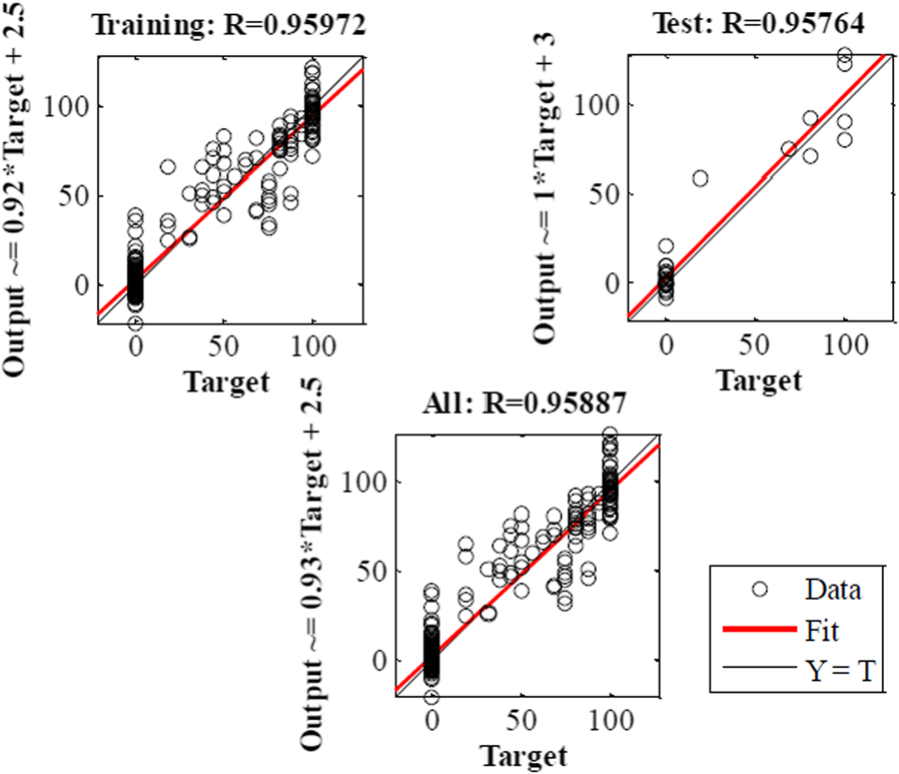
The neural network output regression and the coefficient of multiple correlation (R) for the percentage of callus induction in carrot by MLP model

**Fig. 8:**
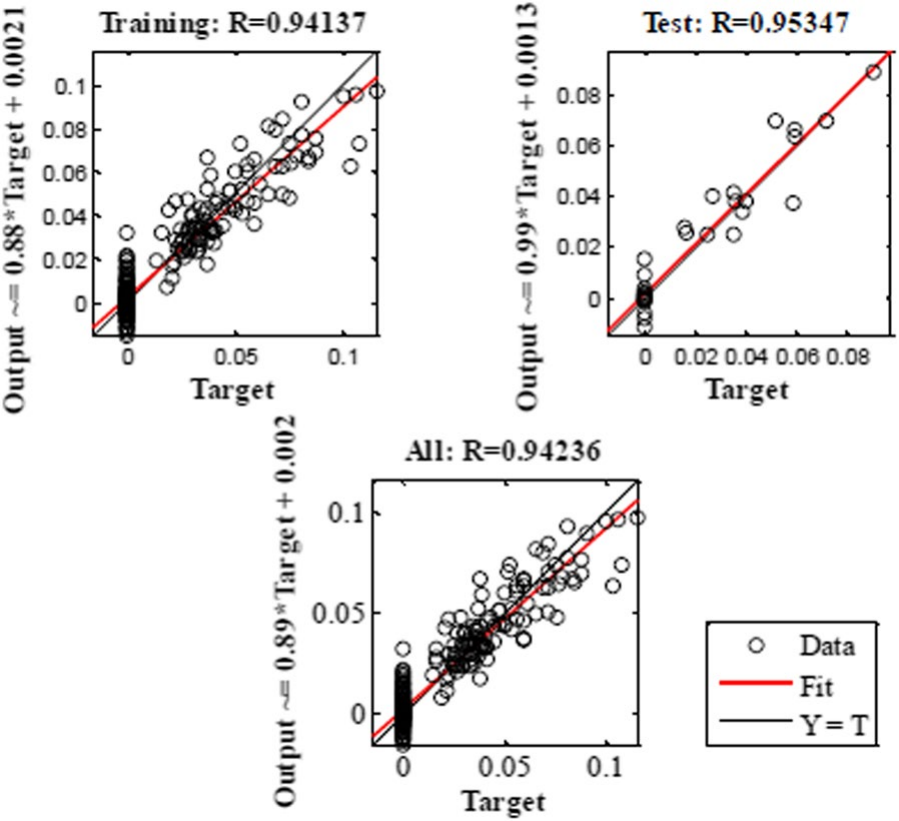
The neural network output regression and the coefficient of multiple correlation (R) for the speed of callus induction in carrot by MLP model

**Fig. 9:**
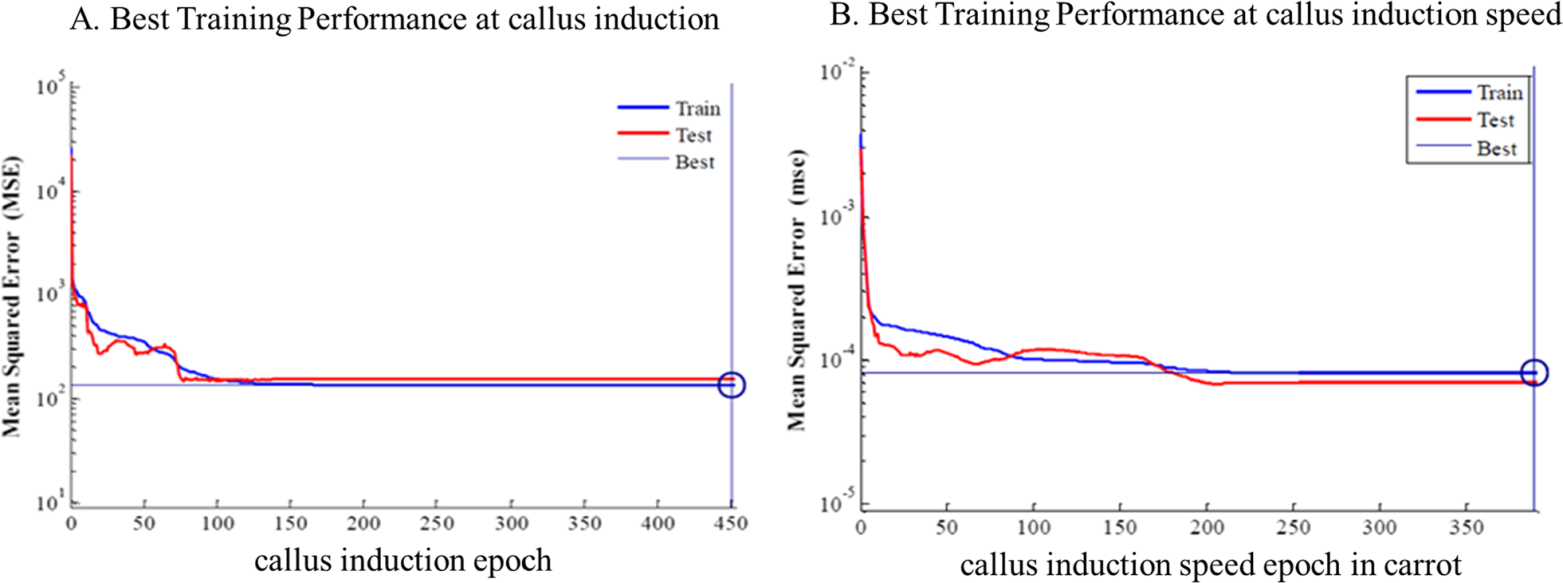
**A** The neural network performance based on MSE vs. epochs of percentage of callus induction in carrot. **B** The neural network performance based on MSE vs. epochs of speed of callus induction

Model accuracy was evaluated by RMSE and MBE, which found MLP to be more accurate than RBF (Tables 1, 2). Also, the regression lines revealed a good fit correlation between experimental and predicted values for embryogenic callus production rate in both the training and testing sets (Figs. 7, 8, 9). Also, the results obtained in the laboratory were confirmed by the MLP model.

### Sensitivity analysis of the models

**Fig. 10:**
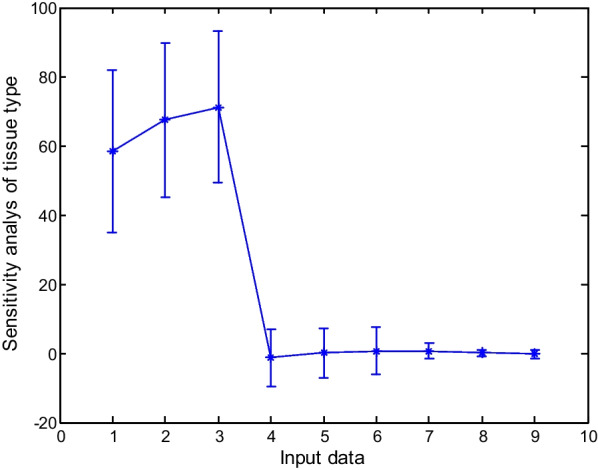
Sensitivity analysis of callus induction based on type of explants in carrot

**Fig. 11:**
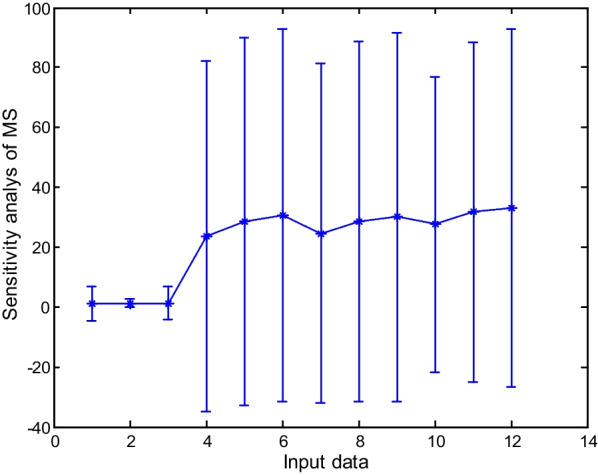
Sensitivity analysis of callus induction based on different concentrations of compound of MS in carrot

**Fig. 12:**
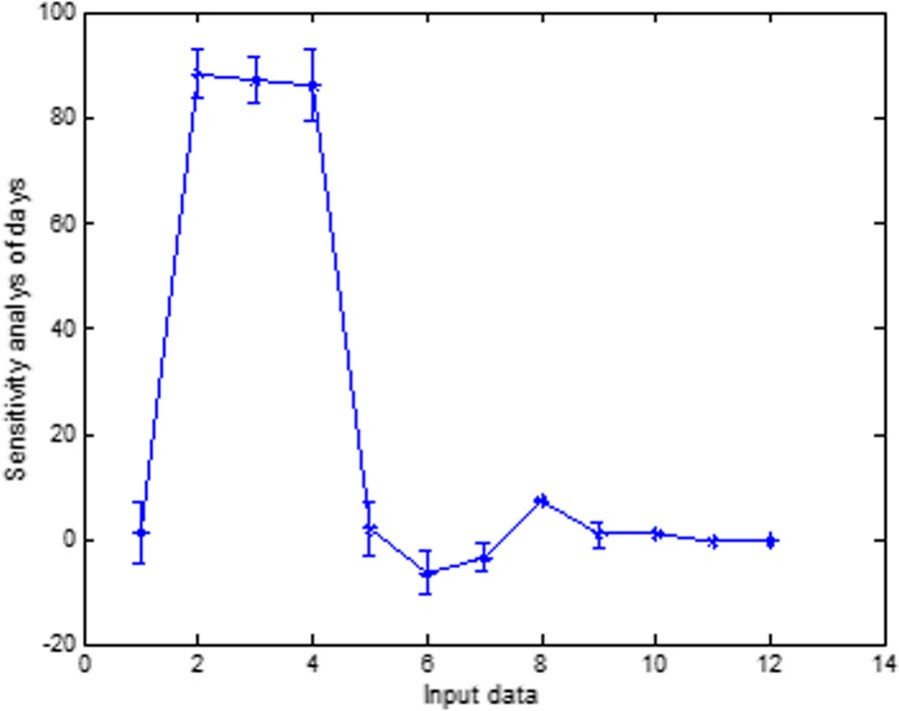
Sensitivity analysis of callus induction based on times of the sampling in carrot

Sensitivity analysis was used to determine the sensitivity values of outputs and the effect of each input data on the percentage and speed of callus induction. The results showed that the highest sensitivity was related to the compound concentration in MS. At the same time, the lowest was related to the time of sampling in outputs, including the percentage and speed of callus induction (Figs. 10, 11 and 12).

### Validation experiment

Based on the results of the validation experiment (Figs. 2, 3, 4, 5, 6), the MLP model was considered an appropriate method to indicate the best explants, the best type of plant, the concentration of plant growth regulators, the time of sampling, and concentration of the compound of MS medium for percentage and speed callus induction. In addition, the MLP model was considered a robust method for predicting the results of in vitro experiments.

In this study, treatment 6 was the best treatment for the percentage and speed of callus induction (Table 3), including 0.5 mg/l 2, 4-D + 0.5 mg/l BAP in shoot explants in 1× MS media. This result was also obtained by laboratory testing and the MLP model.

**Table 3:** The plant growth regulatory used in this study in carrot

Treatment	Plant growth regulators
1	0.3 mg/l BAP + 2 mg/l NAA
2	0.2 mg/l 2,4-D
3	1 mg/l 2,4-D
4	0.2 mg/l 2,4-D + 0.2 mg/l BAP
5	0.2 mg/l 2,4-D + 0.2 mg/l kin
6	0.5 mg/l 2,4-D + 0.5 mg/l BAP
7	1 mg/l 2,4-D + 1 mg/l kin
8	without plant growth regulator

### GA optimization process with MLP model

**Fig. 13:**
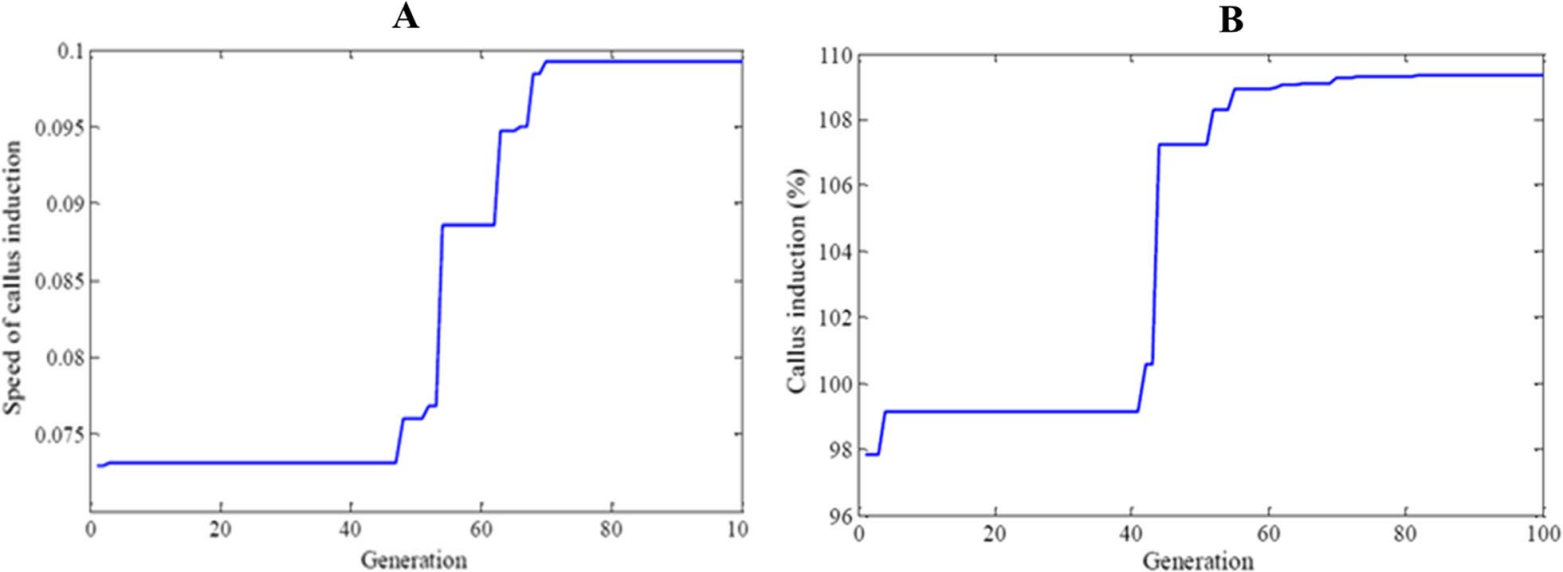
**A** Fitness function of speed of callus induction value vs number of optimization generations in carrot. **B** Fitness function of callus induction percentage value vs number of optimization generations in carrot

**Fig. 14:**
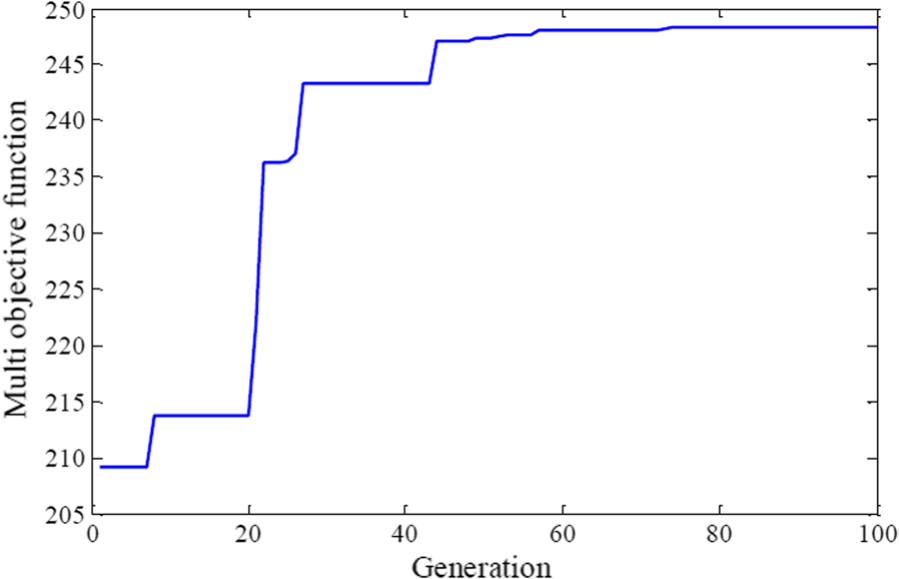
Multi objective fitness function value vs number of optimization generations in carrot

Results of optimizations with different fitness functions are shown in Figs. 13A, B, 14. Considering the speed and the percentage of callus induction, the single, together, and best explants for callus induction are different.

The best fitness function result of the speed of callus induction optimization alone obtain from leaf explants, 1× MS medium on the eighth day of sampling and in the sixth treatment (Fig. 13).

The highest fitness function of the percentage of callus induction optimization alone was obtained from shoot explants, 1× MS medium in 25 days of sampling the sixth treatment (Fig. 13).

Nevertheless, the best fitness function of speed and percentage of callus induction optimization were obtained together from shoot explants, 1× MS medium on the eighth day of sampling, and in the sixth treatment (Fig. 14).

## Discussion

Optimizing tissue culture in plants is essential for manipulation using genetic engineering. The manipulation is used for creating or improving the plant traits and producing vaccines in the plant.

In this regard, callus induction is the first step in the tissue culture process [46], the efficiency of which can be obtained with different explants, types, and concentrations of various plant growth regulators and media. The best speed for each treatment is essential for acquiring the highest callus induction speed.

Testing all the treatments in the lab is complex, time-consuming, and costly. Therefore, it is appropriate to use genetic algorithms to solve these problems.

The results indicated the effect of treatments, including explants, types and concentration of plant growth regulator, time of sampling on concentration, and speed of callus induction via MLP and RBF.

Most plant tissue culture studies have employed MLP for modeling and predicting in vitro culture systems. According to these results, MLP had better performance than RBF for modeling and predicting carrot callus induction.

This study’s experimental results (data obtained from the laboratory) and MLP model results were similar. These results confirmed that the MLP model efficiently predicts tissue culture stages.

In addition, the high correlation between predicted and observed training and testing values indicated that this model is effective for the variables in the study.

One of the factors analyzed in this study was the type and concentration of plant growth regulators. The use of NAA in plant tissue culture was used in some studies [, 7–10, 47–49].

In the present study, analysis of percentage and speed callus induction in sensitivity analysis with MLP model indicates the highest effect in percentage, and speed callus induction was related to 0.5 mg/l 2, 4-D + 0.5 mg/l BAP in shoot explants in 1× MS media. However, a reduction occurred in the percentage and speed of callus induction at low concentrations of BAP and 2, 4-D [47]. These results were confirmed by the data obtained in the laboratory.

In general, 2, 4-D is a type of auxin necessary for inducing callus. Based on the results of the previous study, a high concentration of 2, 4-D can block normal callus induction [50] and disrupt natural genetic and physiological processes [51]. Hardegger et al*.* used 0.1 mg/l 2, 4-D for callus induction in carrot [52]. Also, Marquet-Blouin et al*.* and Yau et al*.* indicated 1 mg/l 2, 4-D as the most influential factor in callus induction in carrot [1, 51]. In another study, Rabiei et al*.* demonstrated 0.2 mg/l 2, 4-D as the highest callus induction [53].

6-Benzylaminopurine (BAP) or benzyl adenine (BA) is a first-generation synthetic cytokinin, which plays a different role in the tissue culture of the plant, including eliciting plants, growth and development responses, setting blossoms, and stimulating fruit richness by stimulating cell division. The results of the present study are consistent with those of Pant et al*.* and Rabiei et al*.*, which indicated 1 mg/l BAP needed for callus induction [12, 54, 55] and Heidegger et al*.*, which used 0.5 mg/l BAP for callus induction. These results are similar to the results of this study [52].

Optimization analysis with the MLP model indicates that MS medium with 1× compound is considered the best medium for callus induction of 4× and 8× MS media. The callus induction in 8× MS media was 0%. Thus, the concentration compound of the medium is essential for the percentage and speed of inducing callus.

In addition, MgSO_4_, CaCl_2,_ and MnSO_4_ are macroelement nutrients essential for growing explants in the tissue culture [8].

Magnesium is an essential component of the chlorophyll molecule, vital for the activity of several nonspecific enzymes, as well as for transferring phosphates. MgSO_4,_ as a chlorophyll molecule, is considered the central atom in the porphyrin structure. A magnesium ion in plants is flexible and plays the role of cation, balancing, and neutralizing anions and organic acids. MgSO_4_ is often used as a unique source of both magnesium and sulfate ions [10].

The concentration of MgSO_4_ is 370 mg/l in MS media. Walker and Sato [56] observed a significant reduction in the callus induction formed from *Medicago sativa* when Mg^2+^ is omitted from the medium. It also indicated the highest level of magnesium in melon, which is effective in callus induction [56].

Additionally, calcium is a cofactor with different enzymes essential for synthesizing cell walls. Calcium deficiency causes shoot tip necrosis. Calcium chloride is the commonly used form of calcium in plant tissue culture. Finally, calcium plays a role in pH cellular, a regulator in the source, as well as the sinking translocation of carbohydrates [57]. In addition, they indicated that 9 mM has the highest effect on callus induction among five concentrations of CaCl_2_ (0.1, 1, 3, 6, and 9 mM). The concentration of CaCl_2_ is 400 mg/l in the MS culture medium.

Manganese (Mn) is a cofactor needed for some enzyme reactions, specifically in respiration and photosynthesis. In addition, manganese sulfate is usually considered the added form in plant medium.

In another study, Sarropoulou et al*.* [58] indicated that the callus induction is doubled at 200 μM MnSO_4_ compared to the control. Further, the concentration of MnSO_4_ is 22.3 mg/l in the MS culture medium.

Eventually, shoot explants, including 1× MS media and 0.5 mg/l 2, 4-D + 0.5 mg/l BAP have the highest effect in percentage and speed callus induction.

## Conclusion

Multiple models should be used to solve the problems related to tissue culture. In this study, MLP-Single point discrete GA, as a new computational tool, was used to predict the percentage and speed callus induction in carrot. This model can be used quickly to identify the interaction between treatments compared to statistical analysis in many experiments. The results indicated that MLP-Single point discrete GA is considered a fit model for predicting the optimum multi-treatment of in vitro tissue culture.

This study used Multilayer Perceptron-Single point discrete GA to optimize in vitro percentage and speed callus induction of carrot for the first time. Accordingly, this is the innovation of this research.

## Supplementary Information

**Additional file 1.** Row data in MS with 8 treatment by using 4 explants at 2 times.

**Additional file 2.** Row data in 4xMS with 8 treatment by using 4 explants at 2 times.

**Additional file 3.** Row data in 8xMS with 8 treatment by using 4 explants at 2 times.

## Data Availability

All data generated or analysed during this study are included in this published article [and its supplementary information files].
