# Supplementary material for: Modeling and optimizing in vitro percentage and speed callus induction of carrot via Multilayer Perceptron-Single point discrete GA and radial basis function
Source: BMC Biotechnol. 2022 Nov 5;22:34. doi: 10.1186/s12896-022-00764-4 (PMC9636657; doi:10.1186/s12896-022-00764-4)
Supplement: Supplementary file 3 — Additional file 3. Row data in 8xMS with 8 treatment by using 4 explants at 2 times. [file 12896_2022_764_MOESM3_ESM.doc]

Row data in 8xMS with 8 treatment by using 4 explants at 2 times
